# Biomembrane-wrapped gene delivery nanoparticles for cancer therapy

**DOI:** 10.3389/fbioe.2023.1211753

**Published:** 2023-06-07

**Authors:** Jie Li, Huamin Zeng, Luwei Li, Ming Song, Mingqing Dong

**Affiliations:** ^1^ Department of Geriatrics, Chengdu Fifth People’s Hospital, Geriatric Diseases Institute of Chengdu, Chengdu, Sichuan, China; ^2^ Center for Medicine Research and Translation, Chengdu Fifth People’s Hospital, Chengdu, Sichuan, China; ^3^ Chengdu Ping An Healthcare Medical Examination Laboratory, Chengdu, Sichuan, China; ^4^ College of Clinical Medical, Chengdu University of Traditional Chinese Medicine, Chengdu, Sichuan, China; ^5^ Department of Pathophysiology, Shenzhen University Health Science Center, Shenzhen, Guangdong, China

**Keywords:** gene delivery, membrane-wrapped biomimetic nanovectors, advantages and obstacles, tumor targeting, efficacy improvement strategies

## Abstract

As a promising strategy, gene delivery for cancer treatment accepts encouraging progress due to its high efficacy, low toxicity, and exclusive selectivity. However, the delivery efficiency, specific biological distribution, targeted uptake, and biosafety of naked nucleic acid agents still face serious challenges, which limit further clinical application. To overcome the above bottleneck, safe and efficient functional nanovectors are developed to improve the delivery efficiency of nucleic acid agents. In recent years, emerging membrane-wrapped biomimetic nanoparticles (MBNPs) based on the concept of “imitating nature” are well known for their advantages, such as low immunogenicity and long cycle time, and especially play a crucial role in improving the overall efficiency of gene delivery and reducing adverse reactions. Therefore, combining MBNPs and gene delivery is an effective strategy to enhance tumor treatment efficiency. This review presents the mechanism of gene therapy and the current obstacles to gene delivery. Remarkably, the latest development of gene delivery MBNPs and the strategies to overcome these obstacles are summarized. Finally, the future challenges and prospects of gene delivery MBNPs toward clinical transformation are introduced. The principal purpose of this review is to discuss the biomedical potential of gene delivery MBNPs for cancer therapy and to provide guidance for further enhancing the efficiency of tumor gene therapy.

## 1 Introduction

Since the 21st century, the tumor is still one of the most severe threats to human life and health. Further research in cancer diagnosis and treatment is an urgent need. At present, chemotherapy is the primary clinical treatment strategy for tumors. However, despite the apparent therapeutic effect, chemotherapy also produces side effects that can not be ignored, such as damage to normal tissues/cells and severe pain, dramatically suffering patients. Therefore, developing treatments that satisfy patients is of great significance for cancer. It requires researchers to understand the essential mechanism of tumors.

Tumorigenesis usually originates from abnormal gene expression or mutation, which regulates tumor cell proliferation, differentiation, apoptosis, and death (Mengzhe et al., 2022). In theory, treating tumors at the genetic level can specifically regulate and modify the abnormally expressed genes in tumor cells and achieve the purpose of curing both the symptoms and root causes. At the same time, gene therapy is regarded as a revolutionary technology for high efficiency, low toxicity, high selectivity, and non-drug resistance (Arjmand and Larjani, 2020). The main nucleic acid molecules utilized in cancer gene therapy are plasmid DNA, small interfering RNA (siRNA), microRNA (miRNA), messenger RNA (mRNA), small hairpin RNA (shRNA), immunostimulatory cytosine-phosphorothioate-guanine oligodeoxynucleotides (CpG ODNs), ribozyme, and gene editing system (e.g., clustered regularly interspaced short palindromic repeats [CRISPR/Cas9]) (Munakata et al., 2019; Damase et al., 2021; Munagala et al., 2021; Tao et al., 2021; Oh and Senger, 2022). To date, gene therapy research is in full swing worldwide, and more than 1,300 projects have entered the clinical trial stage of cancer treatment (Cehajic-Kapetanovic, 2020; Leebeek and Miesbach, 2021). However, naked nucleic acid molecules are volatile *in vivo* and are easily degraded by nucleases, resulting in an off-target effect and poor effect of direct administration (Liu et al., 2007). In order to overcome this bottleneck, researchers have developed various strategies to improve gene therapy efficiencies, such as nuclear transfection, electroporation, and (non) viral vectors (Bucher et al., 2021; Campillo-Davo et al., 2021; Zu and Gao, 2021; Qi et al., 2022). Unfortunately, nuclear transfection and electroporation will cause irreversible damage to cells, and traditional vectors are challenging to cope with the test of the complex tumor microenvironment (TME), so their clinical application is limited.

Recently, with the in-depth exploration and development of biomimetic materials, biomimetic nanoparticles (NPs) have attracted wide attention and application in gene delivery for tumor therapy (Liang et al., 2022; Cai et al., 2023). Different from the traditional vectors, biomimetic NPs are endowed with more functions and advantages through the careful modification of biomolecules, such as biomembranes (Cardellini et al., 2022; Huang et al., 2023d), protein macromolecules (Nierenberg et al., 2022), nucleic acid aptamers (Ma et al., 2022). Among them, membrane-wrapped biomimetic nanoparticles (MBNPs) are a promising approach to evade immune clearance. The MBNPs miraculously escape the reticuloendothelial scavenger cells compared to the conventional non-viral vectors. For example, the single biggest hurdle in systemically administered gene delivery vectors is nonspecific capture by the liver scavenger sinusoidal wall cells, causing a substantial decrease in the delivery efficiency of gene delivery vectors into the target tissues (Dirisala and Uchida., 2020). In contrast, MBNPs evade this scavenger immune cell recognition by presenting their surface “don’t recognize” and “don’t scavenge me” signals that prevent them from being marked as foreign (Sushnitha et al., 2020). They provide the advantages of better biocompatibility, improved drug loading capacity, better cell interaction, and reduced toxicity *in vivo*, which makes it reflect better biosafety and efficacy in the application (Harris and Sterin., 2022; Yang et al., 2023). In addition, MBNPs can accurately deliver drugs to the target tissue and prevent drug removal, degradation, or release prematurely (Zhang et al., 2022c; Wu et al., 2022). Therefore, MBNPs overcome the limitations of traditional vectors and are widely used as a delivery system to accurately transmit gene agents to the focus, thus maximizing the efficiency of gene therapy.

**FIGURE 1:**
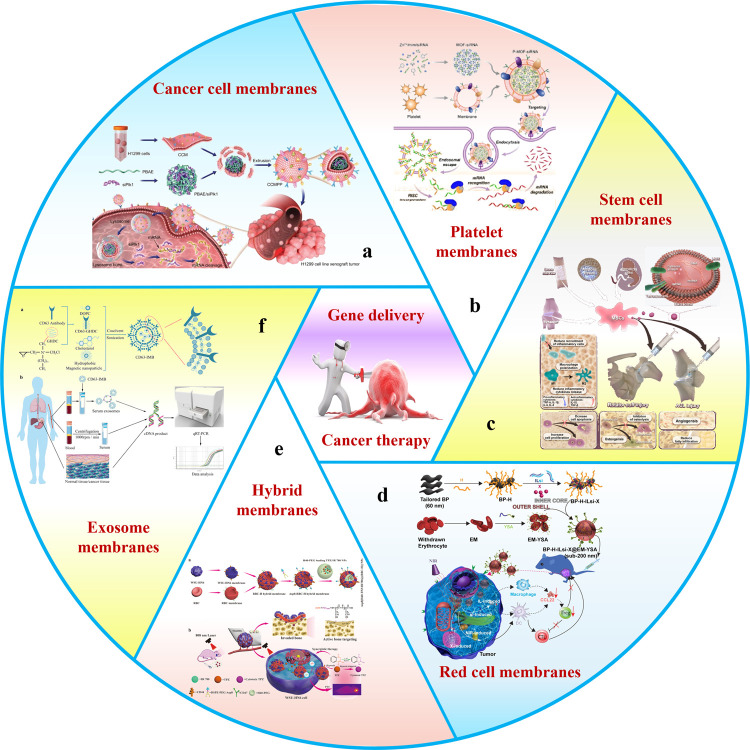
Schematic of membrane-wrapped biomimetic gene delivery nanoparticles for cancer therapy. This schematic includes several biomembrane: **(a)** cancer cell membranes, **(b)** platelet membranes, **(c)** stem cell membranes, **(d)** red cell membranes, **(e)** hybrid membranes, and **(f)** exosome membranes.

By far, the research on the combination of biofilm and nano-vectors has been more and more in-depth. The emergence of MBNPs provides an epoch-making strategy and lays a foundation for the clinical transformation of tumor gene therapy. The biofilms used for MBNPs mainly include cancer cell membranes (CCMs) (Chen et al., 2019a), red cell membranes (RCMs) (Zhang et al., 2022c), stem cell membranes (SCMs) (Ho and Kim, 2021), platelet membranes (PMs) (Wang et al., 2019), exosome membranes (EMs) (Wang et al., 2022), bacterial membranes (BMs) (Chen et al., 2022a) and hybrid membranes (HMs) (Chen et al., 2020a; Zhao et al., 2021) (Figure 1). Considering biofilm’s unique advantages, this review’s principal purpose is to classify and summarize the research progress of various gene delivery MBNPs for cancer therapy to enhance the efficiency of tumor gene therapy. Moreover, the biomedical potential of transforming MBNPs into clinical applications will be deeply tapped.

## 2 The mechanism and present situation of gene delivery

Although cancer treatment programs have improved over the past few decades, the standard anti-cancer method still depends on a combination of surgery, radiotherapy, and chemotherapy. However, the clinical practice faces severe challenges due to the high side effects, poor selectivity, high tumor recurrence rate, and drug resistance. Remarkably, a new strategy, gene silencing, has been proposed to curb the deterioration and proliferation of tumors in terms of gene expression, which is regarded as one of the most promising treatments in anti-tumor research (Chen et al., 2019b; Nuñez et al., 2021). Studies have found that cancer cell proliferation will be inhibited when cancer-related genes are expressed abnormally. Specifically, exogenous nucleic acid molecules (For details, see the Section 1) are introduced into tumor cells to correct abnormal genes or compensate for defective genes, thus increasing or reducing the expression of target proteins to achieve treatment purposes (Vetvicka et al., 2021). After the abnormal expression of cancer-related genes, the nutritional metabolism in cancer cells can not be carried out typically, so it can not continue to grow and proliferate. Gene silencing can selectively control cell-related enzymes and organelles, regulate intracellular energy metabolism, and owns a bright application prospect in tumor therapy. In August 2018, the US Food and Drug Administration approved the first siRNA nano-drug Patisiran (ONPATTRO) (Akinc et al., 2019). This milestone brought gene therapy from basic research to clinical application. Patisiran represents a breakthrough in nucleic acid therapy based on RNA interference and nanotechnology, and opens a new era of rapid development of gene nanomedicine therapy.

## 3 Barriers to gene delivery

Delivery efficiency is the key in gene therapy, meanwhile, the need for more safe and effective delivery means is the bottleneck to limiting its clinical application. Nano-vectors for gene delivery face numerous physiological barriers before reaching focus: i) clearance by the reticuloendothelial system (RES); ii) insufficient accumulation of tumor sites mediated by enhanced permeability and retention (EPR) effect; iii) deep penetration obstruction of drugs into tumor tissues; vi) low uptake efficiency by tumor cells; and v) early release or degradation of nucleic acid molecules. The above five steps substantially impact gene delivery efficiency, which should be considered in all aspects when designing nano-vectors.

### 3.1 Identification and removal of RES

In order to successfully deliver genes to target cells, nano-vectors need to overcome various interference factors due to the complex environment *in vivo*. The most conventional gene delivery vector construction strategy is to closely compress negatively charged nucleic acid molecules and positively charged nanomaterials by electrostatic adsorption to form complexes (Qiu and Tang, 2022). The positive charge can promote the uptake of negatively charged cell membranes, however, it also inevitably leads to non-specific interaction between complex and plasma protein, cells, and intercellular stroma, so it is easy to be recognized and cleared by RES during circulation (Pattipeiluhu and Arias-Alpizar, 2022). This process makes it difficult for nano-vectors loaded with nucleic acid molecules to reach the tumor site, a significant hurdle affecting the efficiency and distribution of gene delivery *in vivo*.

### 3.2 Heterogeneity of EPR effect

The vascular endothelial space of normal tissue is very dense and intact, approximately 5–10 nm, so it is complicated for macromolecules to penetrate the vascular wall. Inversely, tumor tissue exists abundant abnormal blood vessels and incomplete vascular wall structure, by which the arrangement of tumor vascular endothelial cells is sparse and permeable, with the pore size between 100 and 300 nm, because the growth rate is significantly higher than that of normal tissue. Simultaneously, the internal characteristics of the tumor are insufficient lymphatic drainage and low blood flow rate. Therefore, once the NPs enter a tumor’s blood vessels, they will be trapped in the focus site. The phenomenon, known as the EPR effect (Liu et al., 2019), is generally considered the main reason NPs accumulate in tumor sites. Based on this, the optimum size range of nano-vectors for gene delivery is about 100–200 nm, which is conducive to the passive targeting of NPs. However, the gap between endothelial cells, one of the leading factors of the EPR effect, only occurs occasionally (Sindhwani and Syed, 2020). The total coverage rate is only 0.048% of the vascular surface area; the actual number is 60 times less than the theory. Hence, the EPR effect has been questioned recently. Sufficient evidence shows that the trans endocytosis of endothelial cells may be the critical mechanism for NPs accumulating in the tumor site, and the discovery may provide a new idea for the design of nano-vectors.

### 3.3 Cellular endocytosis process

**FIGURE 2:**
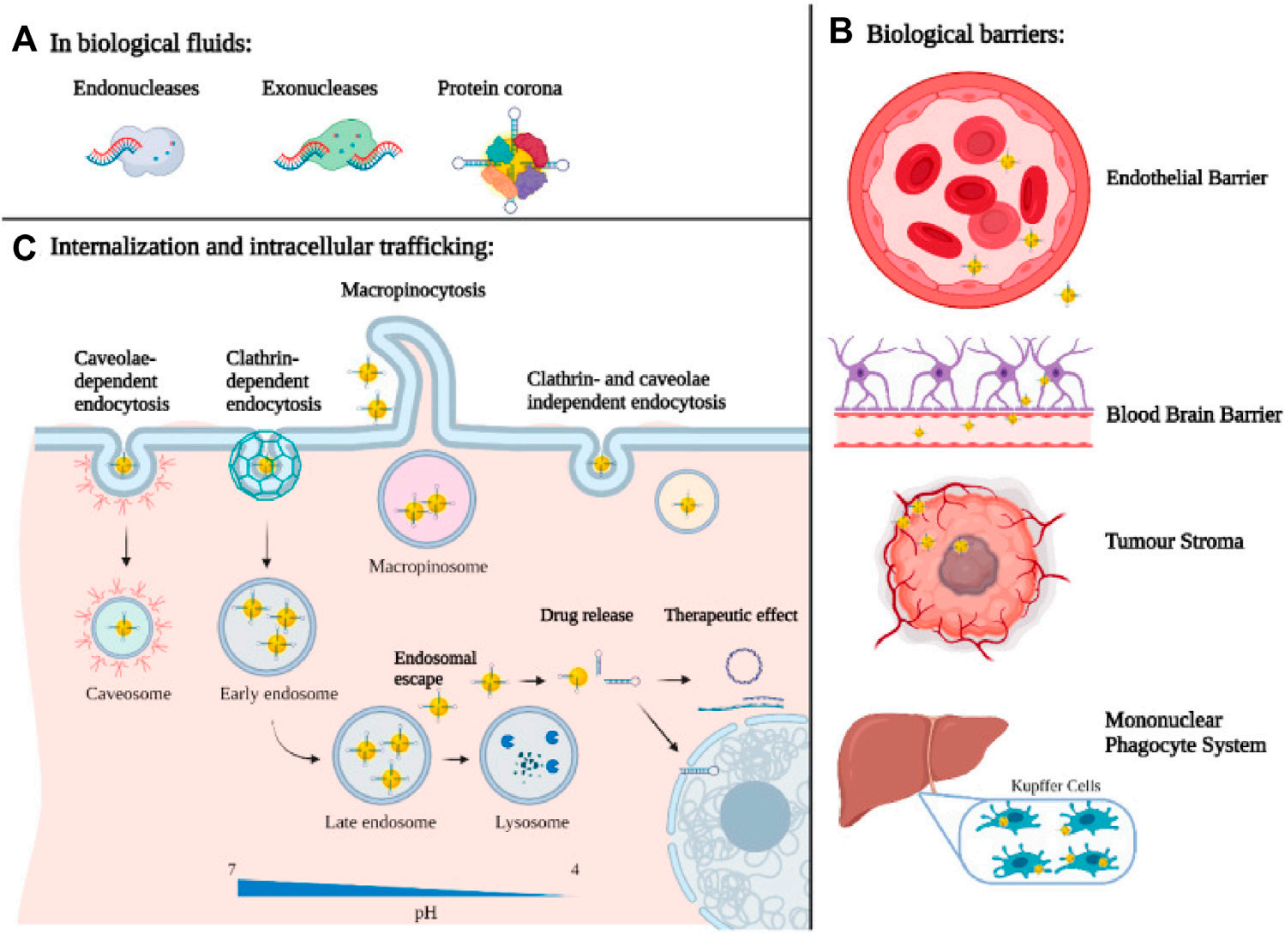
Biological barriers for nanotechnology-based gene delivery systems: **(A)** at the organ/tissue level, **(B)** at the molecular level **(C)** and at the cellular level (Mirón-Barroso et al., 2021).

One of the essential requirements for the utilization of non-viral vectors in tumor therapy is to specifically target the focus site to avoid an off-target effect, which could achieve by binding tumor proteins (ligands) to NPs, for that there are overexpressed receptors in tumor cells (e.g., transferrin receptor, folate receptor, CD44 receptors, and epidermal growth factor receptor) (Candelaria et al., 2021; Nallasamy et al., 2021; Trivedi and Ferris, 2021; Nawaz and Kipreos, 2022). After ligand-receptor binding, targeting NPs enter tumor cells mainly through endocytosis-mediated internalization. Generally speaking, the standard internalization mode includes phagocytosis (Butler and Popescu, 2021), clathrin-mediated endocytosis (Moreno-Layseca and Jantti, 2021), caveolae-mediated endocytosis (Nie et al., 2021), lipid raft-mediated endocytosis (Zhang et al., 2021c), micropinocytosis (Zhang et al., 2022b) (Figure 2). Previous studies have shown that caveolin-mediated endocytosis can transport nucleic acids directly to the Golgi apparatus or endoplasmic reticulum via the endosome-golgi apparatus-endoplasmic reticulum pathway, thus avoiding degradation in lysosomes (Wang et al., 2020a). In addition, other forms of endocytosis are not conducive to the gene delivery of NPs. Therefore, it is necessary to design personalized internalization methods for different gene delivery systems. Different endocytosis pathways can be endowed to nano-vectors by structural adjustment and surface modification, which helps improve gene delivery efficiency.

### 3.4 Endosome/lysosome capture

After entering the cells, most non-viral vectors can not escape the misfortune of being captured by endosomes/lysosomes, resulting in the degradation of foreign genes by acid hydrolases. Therefore, whether the nucleic acid molecules delivered into cells can escape from endosomes/lysosomes is an incontrovertible core issue affecting gene transfection efficiency. There are three principal methods of endosome/lysosome escape: i) reduces the stability of the lysosome membrane by electrostatic action. Both cationic liposomes and cationic polymers exhibit the ability to induce endosome/lysosome rupture and nucleic acid molecule release (Wu et al., 2023). ii) disturbs the stability of lysosomes by changing pH. Lysosome escape can be achieved by adding reagents to promote lysosome ruptures, such as chloroquine, sucrose, and polyvinylpyrrolidone (Beach et al., 2022). iii) destroys the structure of the endomembrane by producing reactive oxygen species (ROS) (Postiglione and Muday., 2020). Photochemical internalization has become an attractive strategy to help nucleic acids escape from endosomes. The ROS produced by photosensitizers coated in nano-vectors can promote vectors’ escape from endosomes/lysosomes and destroy polymers to release DNA, thus achieving effective gene delivery (Soe and Watanabe, 2020).

### 3.5 Release of nucleic acid molecules

Non-viral vectors can play a role only if they carry foreign nucleic acid molecules to specific targets. Different nucleic acid molecules achieve gene therapy at different sites. RNA molecules perform their therapeutic function only need to reach the cytoplasm, while DNA must be transferred to the nucleus. One of the keys to the non-viral gene delivery pathway is to release complete nucleic acids from NPs to the cytoplasm or nucleus of target cells (Gantenbein et al., 2020; Zu and Gao., 2021; Yan et al., 2022). If released too slowly or not, nucleic acid molecules will not be transcribed and will eventually lose through exocytosis and mitosis. Oppositely, when the release is too easy, or the nano-vectors are not tight enough to resist the penetration of the enzyme, the nucleic acid molecule can quickly degrade before reaching the target site. So, the ideal non-viral gene vectors should closely compress the nucleic acid molecule, protect it from enzyme degradation before reaching the target cell, and release agents in time for follow-up transfection (Li et al., 2015). However, the research on non-viral vectors has yet to effectively solve the above obstacles in the process of gene delivery, and how to improve the delivery efficiency is still a core problem to be solved urgently.

## 4 Application of MBNPs

### 4.1 History of membrane-wrapped technology

Since 2011, Hu et al. (2011) have developed a top-down biomimetic method, named the membrane coating technique, in which they used natural red cell membranes to encapsulate polymer NPs for the first time to reduce macrophage uptake and systemic clearance. This method provides a new solution to the above problems. Later (in 2013), Parodi et al. (2013) focused on the selection of membranes on nucleated cells and used white blood cells as a source of membrane materials. In 2015, Hu et al. (2015) enriched the raw materials of membrane coating and shifted the focus of research from normal human cells to cancer cells and bacterial cells, and applied mitochondrial membrane to the field of cell membrane biomimetics in 2021 (Gong et al., 2021b). In addition, there are reports and studies on using stem cells, macrophages, neutrophils, T lymphocytes, natural killer cells, and dendritic cells in the cell membrane biomimetic field. Even some MBNPs have been considered for clinical applications.

**FIGURE 3:**
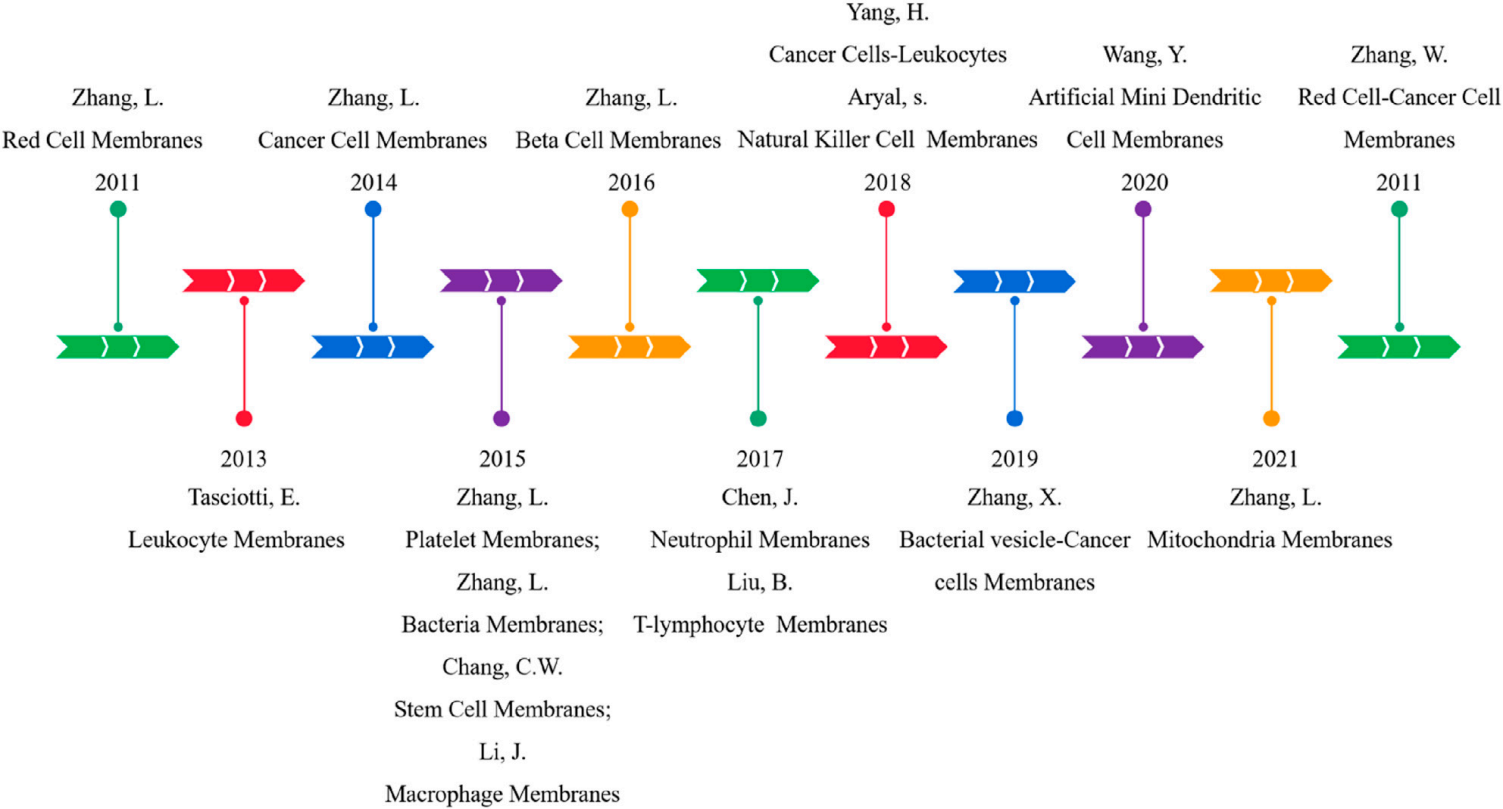
Development schedule of CMC-NPs.

In order to further integrate various functions, Dehaini et al. (2017) first proposed hybrid cell membrane-coated nanoparticles as early as 2017. Red blood cell-platelet NPs preserve proteins from parent cells, combining their unparalleled functions. Subsequently, more and more functional cells are combined to modify NPs, such as cancer cell-red cells, macrophage-cancer cells, and bacterial vesicle-cancer cells (Chen et al., 2020a). Double-cell membrane NPs are the most commonly used hybrid biomimetic particles. However, only some researchers have used mixtures of three or more membranes because the preparation and testing processes are too tanglesome. The development schedule for MBNPs is shown in Figure 3.

### 4.2 Preparation method of MBNPs

**FIGURE 4:**
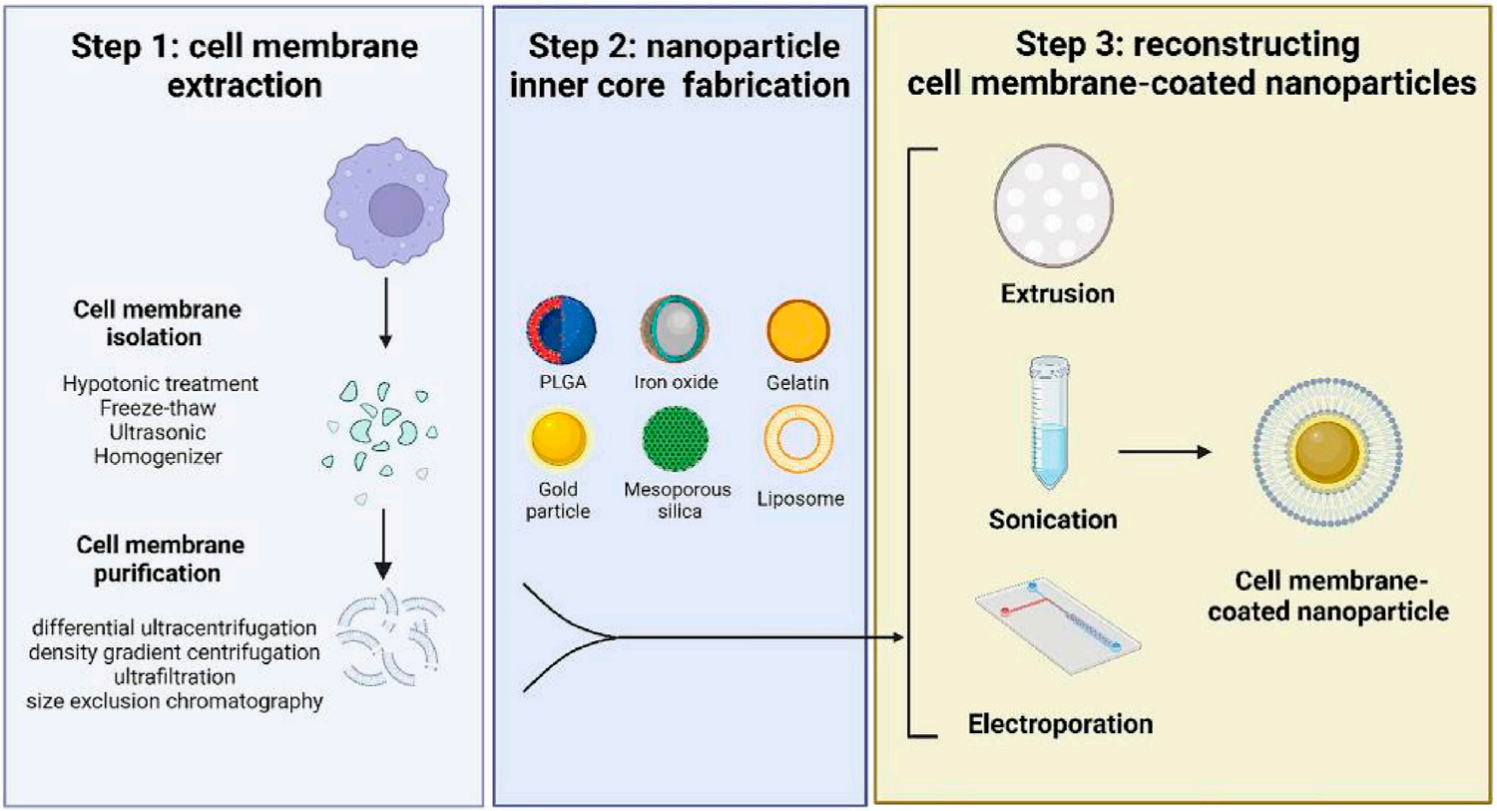
A schematic diagram of preparing cell membrane-coated nanoparticles. Step 1 includes two processes of harvesting cell membrane fragments; Step 2 requires cautious selection and fabrication of the inner core according to the purpose; Step 3 is the final step to coat the cell membrane onto a template (Liu et al., 2023).

Generally, the preparation of MBNPs consists of three steps (Figure 4), namely, cell membrane extraction (step 1), NPs kernel fabrication (step 2), and MBNPs construction (step 3). Methods extensively utilized for membrane isolation (e.g., hypotonic lysis, freeze-thawing, ultrasound, and homogenization) and membrane coating (e.g., physical extrusion, sonication, and microfluidic electroporation) are listed in Table 1 (Liu et al., 2023). NPs kernel manufacturing is not discussed much in this article.

**TABLE 1:** Comparison between kinds of preparation method of MBNPs.

Types		Advantages	Disadvantages
Cell membrane extraction	Hypotonic lysis	can become swollen and rupture under low osmotic pressure; widely used in erythrocyte membrane extraction but	Is not commonly used in the extraction of other cells because of its low efficiency
	Freezethawing	are frozen at low temperatures and repeatedly thawed at room temperature; with high extraction efficiency of platelet membrane extraction	Freezing and thawing can partially affect protein activity
	Ultrasound	Ultrasonic waves cause cell breakage; high extraction efficiency; suitable for most microorganisms	Generates large amounts of heat
	Homogenization	Can shear cells into smaller pieces and disperse them; is suitable for fragmentation of various cell types; potential application in large-scale industries	Belongs to energy intensive strategy; causes a heavy maintenance workload; is poorly suited for samples with high viscosity
Construction of MBNPs	Physical extrusion	Better uniformity and smaller size dispersity	Abundant waste for the porous membrane
	Sonication	Can prevent material loss and yield a high degree of dispersion	May cause uniformity and an uneven size
	Microfluidic electroporation	High throughput and quantitative format; scalability and storage capacity; may be used at industry scale	The lack of specifications and standards for core technologies need to be addressed

Step 1: Membrane extraction consists of membrane separation and purification, which process requires a delicate operation to maintain the structural integrity and purity of the membrane. It is beneficial to maximize the functionality preservation of the parent membrane and minimize adverse reactions. Similarly, hybrid membranes can also be prepared by ultrasound or extrusion. This method is suitable for membrane hybridization of most nucleated and non-nucleated cells. Subsequently, membrane purification by differential ultra-centrifugation, density gradient centrifugation, and ultrafiltration can further improve the performance of MBNPs. Step 2: NPs cores were prepared according to the requirements for that different kernels endow unique properties to MBNPs. There are two main types of kernels: inorganic cores (e.g., gold NPs, Fe_3_O_4_ NPs, graphene NPs) and organic cores (e.g., gelatin, liposome, and poly (lactic-co-glycolic acid) [PLGA]). The encapsulation of cell membranes avoids the direct contact between NPs and the internal environment, thus giving them higher biocompatibility and great potential for safe and effective therapy. Step 3: The construction of MBNPs. In general, membrane coverage, functional efficiency, particle size, and dispersity are essential factors to be considered when selecting a coating method.

### 4.3 Classification of MBNPs

Clearly, the naked nucleic acid molecule is volatile and easily rapidly degraded by nuclease in the circulation process *in vivo.* Simultaneously, with the high renal clearance and low uptake efficiency, serious side effects from off-target effects are still a rigorous challenge. Even, most of the

reported non-viral vectors have failed to overcome the above dilemmas, so the frustrating transfection efficiency severely limited their clinical application. Therefore, it is necessary to choose a safe and stable gene vector to deliver nucleic acid agents to the target tissue/cells. The ideal gene vector for tumor therapy should unfold excellent stability and non-recognition by RES *in vivo* circulation, and the nucleic acid could be released timely and efficiently when it reaches the tumor site. Fortunately, MBNPs emerge at a historical moment and can meet the above properties simultaneously: i) protect nucleic acid molecules from degradation and premature release; ii) penetrate into tumors, reach tumor cells far away from blood vessels and internalize to improve transfection efficiency; iii) stable and safe in blood circulation. In addition, the application of MBNPs can reduce the toxicity of various inorganic and organic NPs and play an irreplaceable role in maintaining endogenous environment safety. In short, MBNPs exhibit significant advantages against traditional stealth gene vectors, such as PEGylated polyplexes and lipoplexes, for that MBNPs do not induce such a low cellular uptake and transfection dilemma. On the contrary, surface shielding of gene delivery vectors using PEG dramatically decreases cellular uptake and, thereby, transfection efficiency, although PEG improves blood circulation times (termed as PEG dilemma) (Dirisala et al., 2014). MBNPs have been widely studied because of their low immunogenicity, long cycle time, and good biocompatibility. Therefore, the gene delivery approach based on MBNPs is the key to expanding the application of gene silencing technology in tumor therapy. The parent membrane sources, including CCMs, RCMs, SCMs, PMs, EMs, BMs, and HMs, are summarized as follows (Wang et al., 2020b; Huang et al., 2023b).

#### 4.3.1 Cancer cell membranes

**FIGURE 5:**
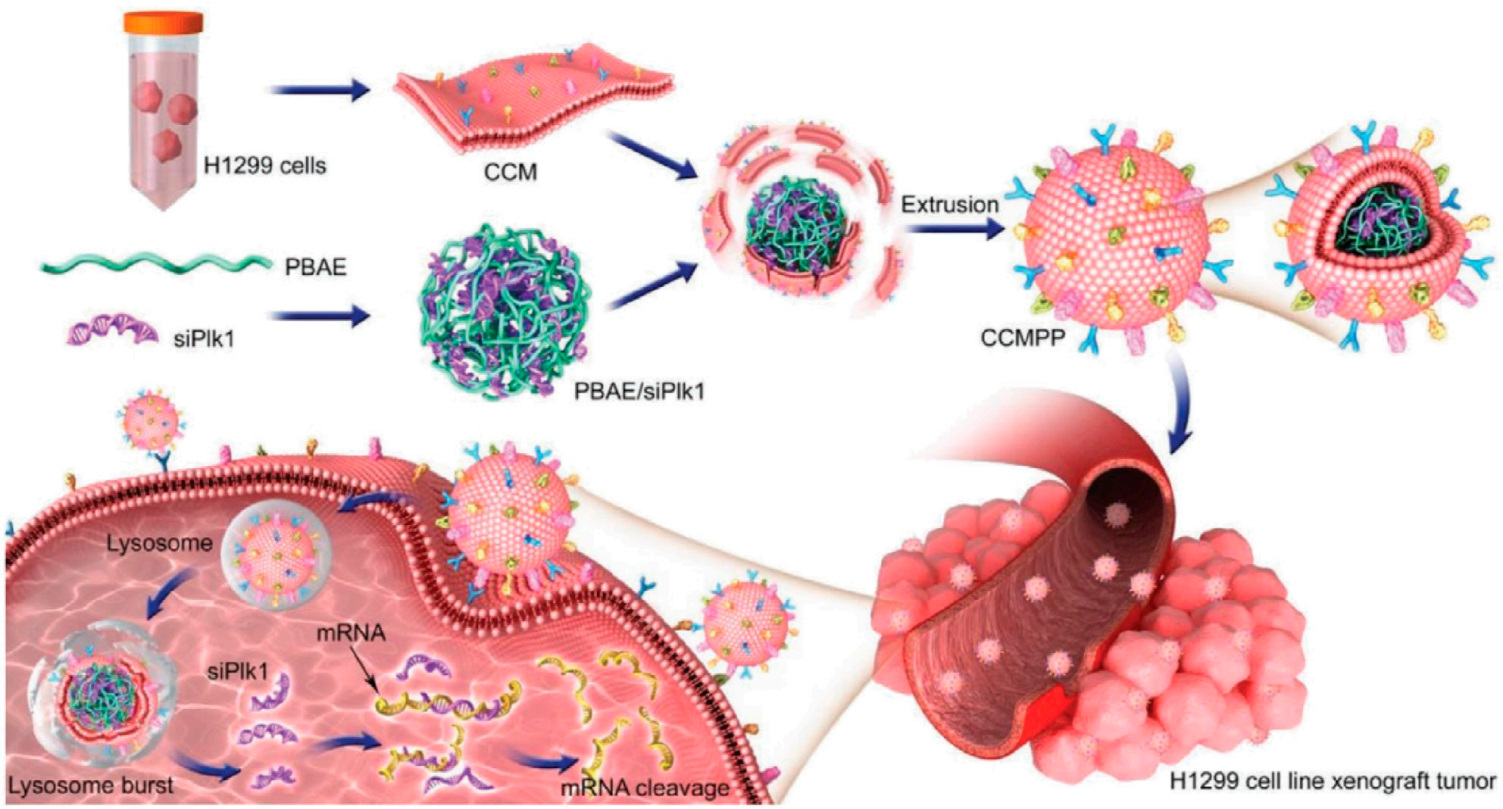
The schematic diagram of the preparation of CCMs-derived MBNPs and the cellular uptake (Zhang et al., 2020).

CCMs-camouflaged NPs are one of the common biomimetic gene delivery systems. Due to the mediation of “self-recognition molecules” on the cell surface and the transfer of cell adhesion molecules with homotype binding characteristics, CCMs own a high affinity for parent cells, called homologous targeting. Therefore, the CCMs-derived MBNPs are endowed with the functions of extravasation, chemotaxis, and adhesion, which can improve the tumor-specific transmission of nucleic acid molecules. In particular, compared with RCMs-derived MBNPs, the uptake of CCMs-derived MBNPs increased by 20 times. The unique characteristics come from the membrane surface biomarkers, such as N-cadherin, galectin-3 or epithelial cell adhesion molecule (Zhu et al., 2016). For example, Chen et al. (2019a) created CCMs-derived MBNPs based on PLGA NPs (CCMNPs) for co-loading doxorubicin (DOX) and PD-L1siRNA. The results showed that CCMNPs possessed higher uptake and cytotoxicity in homologous tumor cells than that of others, proving the specificity of membrane coating. In addition, CCMNPs perform more muscular gene silencing effects on PD-L1 in tumor cells than PLGA NPs. Similarly, Zhang et al. (2020) constructed CCMs-derived MBNPs (CCMs/PBAE/siPLK1) with CCMs via the poly (β-amino ester) core to achieve siPLK1 delivery (Figure 5). Compared with the control group, CCMs/PBAE/siPLK1 existed particular targeting effects on homologous cancer cells and effectively improved the delivery efficiency of siPLK1. Based on this, the level of PLK1 in cancer cells was significantly downregulated, showing a significant tumor inhibitory effect. In sum, these results confirm that CCMs-derived MBNPs promote the effective delivery of siRNA and accurate treatment of tumors.

#### 4.3.2 Red cell membranes

RCM is another effective biomimetic material for tumor gene delivery, which is widely used in constructing biomimetic NPs because of its unique structure, surface protein and function (Chen et al., 2022b). It is well known that the surface markers of red cells, such as glycoprotein and CD47 (CD47 displayed on the RCM acts as a “don’t eat me” signal to phagocytes via CD47-SIRPα signaling (Rodriguez et al., 2013)), can protect NPs from immune surveillance, reduce RES uptake and immunogenicity, thus prolonging blood circulation time and reducing immune clearance rate. In addition, the RCMs-derived MBNPs can also neutralize biological and chemical toxins. Yang et al. (2019) prepared RCMs-derived MBNPs for miRNAs analysis, which can not only achieve one-step quantification of miRNAs in complex media, but also does not need the process of enzymatic amplification, displaying a certain degree of anti-interference. The NPs perform ultra-sensitive quantitative ability and high selectivity for miR-141/200 family members. More importantly, the NPs can accurately screen patients with lung or prostate cancer, and achieve rapid and sensitive early diagnosis, showing great potential in clinical diagnosis. Xu et al. (2022) developed a simple RCMs-derived MBNPs to co-delivery of siRNA and chemotherapy drugs. Molecular dynamics simulation experiments show that RCMs-derived MBNPs can significantly improve the physiological stability of siRNA and prolong its circulation time *in vivo*, performing well in treating drug-resistant cancer, which provides a safe and effective way to apply gene combined chemotherapy in multidrug resistant tumors. However, due to the heterogeneity of RCMs, the RCMs-derived MBNPs own no favorable targeting properties for tumors.

#### 4.3.3 Stem cell membranes

It is reported that SCM is regarded as a promising biomimetic material because of its excellent tumor targeting and homing ability, which is mainly attributed to the interaction between the highly expressed CXCR4 on SCMs surface and the chemokine matrix derived factor 1 (SDF-1/CXCL12) released by tumor cells (Khare et al., 2021). For this, SCMs-derived MBNPs have aroused widespread interest in gene delivery for cancer therapy.

Although PD-L1siRNA can downregulate the level of PDL1 and restore the immune anti-tumor activity of T cells, the therapeutic effect of single immune checkpoint blockade is relatively deficient. Interestingly, DOX can induce tumor cell apoptosis and increase the release of tumor antigen, but it can adaptively upregulate the expression of PDL1. Therefore, the combined application of DOX and PD-L1siRNA can produce an excellent synergistic anti-tumor effect. Accordingly, Mu et al. (2021) constructed SCMs-derived MBNPs camouflaging dopamine (PDA-DOX/siPD-L1@SCMs) to co-loaded with DOX and PD-L1siRNA for the treatment of bone metastasis associated with prostate cancer. The entrapment of SCMs can effectively improve the blood circulation time of NPs and the targeted accumulation in tumor sites, performing an efficient delivery efficiency of PD-L1siRNA.

Mesenchymal stem cells (MSCs) can be recruited explicitly into TME under the action of growth factors and cytokines secreted during tumor angiogenesis and matrix formation (Zou et al., 2023). Therefore, MSCs express a powerful blood circulation capacity and vigoroso tumor targeting ability, thus inducing its drug delivery ability in TME. Therefore, the MSCs membrane is regarded as a biomimetic material to increase the gene delivery efficiency of tumor therapy in TME. For illustrate, in order to enhance the delivery efficiency of nucleic acid drugs, Ho and Kim. (2021) loaded Cas9/sgRNA lipid NPs and chemokine CXCL12α onto MSCs membranes encapsulated nanofiber scaffolds (MSCs-Cas9RNPs) that mimic bone marrow microenvironment to treat acute myeloid leukemia. Under the action of MSCs, CXCL12α releases and induces leukemic stem cells to migrate toward scaffolds, and MSCs-Cas9RNPs induce effective gene editing. At the same time, MSCs increase the retention time of MSCs-Cas9RNPs in the bone marrow cavity, providing an effective strategy for treating acute myeloid leukemia.

#### 4.3.4 Platelet membranes

**FIGURE 6:**
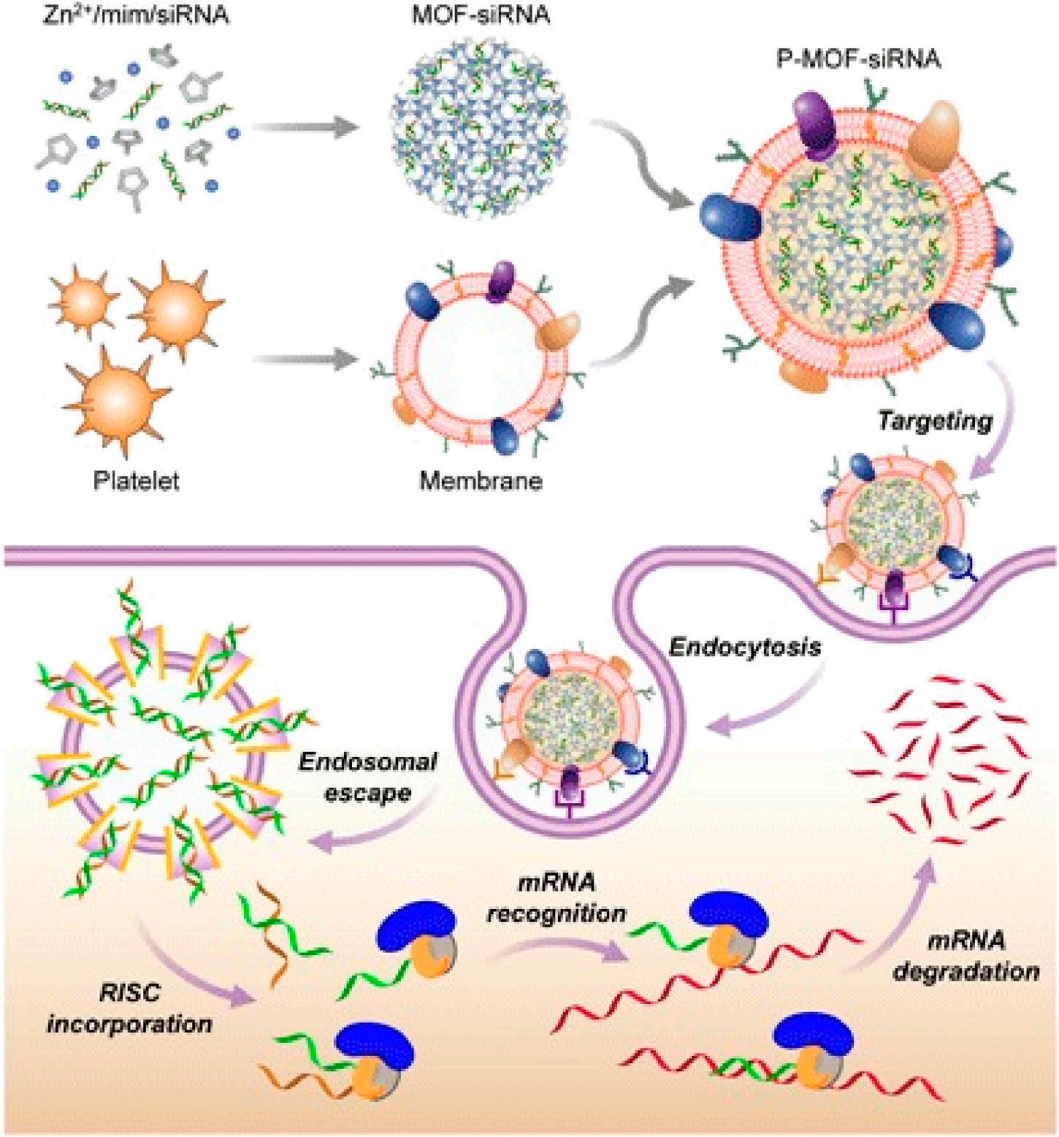
PMs-MOF-siRNA for gene silencing. To fabricate PMs-MOF-siRNA, siRNA-loaded MOF cores are developed by mixing the siRNA payload with 2-methylimidazole and Zn^2+^ based on PMs. When PMs-MOF-siRNA NPs are endocytosed by cancer cells, the low pH induces escape of the siRNA into cytoplasm. mRNA is recognized and degraded, leading to gene silencing by incorporating with RNA-induced silencing complex (Zhuang et al., 2020).

PM is another biomimetic material of great concern, attributed to membrane proteins, such as p-selectin and CD47, which enable platelets to target damaged blood vessels and cancer cells (Wang et al., 2019). In addition, PMs can also protect NPs from immune system attacks and promote the binding of NPs to damaged blood vessels or specific pathogens. More importantly, the extracting and purifying process of PMs is relatively simple, which is beneficial to the clinical transformation and application of PMs-derived MBNPs in cancer therapy. Activated PMs-derived MBNPs have been demonstrated to target and adhere to microthrombus associated with circulating tumor cells in the vascular system, to deliver drugs efficiently and prevent tumor cell metastasis and spread. Therefore, PMs-derived MBNPs, as a nucleic acid delivery vector, possess great potential in tumor gene therapy. For example, Zhuang et al. (2020) reported PMs-derived MBNPs for targeted delivery of siRNA *in vivo* (Figure 6). The results showed that the PMs coating existed blatant anti-tumor targeting and high silencing efficiency of multiple target genes *in vitro* (40%–80%). In addition, the encapsulation of PMs can also improve the therapeutic sensitivity of tumors. Chen et al. (2021b) developed a kind of gold PMs-derived MBNPs, which can not only avoid being cleared by the immune system, but also actively target drugs to kill tumor cells, reducing resistance to anti-tumor effects. Under the action of PMs-derived MBNPs, tumor treatment becomes more effective.

#### 4.3.5 Exosome membranes

The exosome is a kind of endogenous extracellular vesicle (40–100 nm), which is mainly secreted into the extracellular matrix by the internal chamber of eukaryotic cells, playing an essential role in cell-to-cell information transmission (Zhang et al., 2021b). Exosomes are abundant in various cancer cells and can carry unique nucleic acid molecules freely through the body, which are proved to equip higher delivery efficiency, lower immunogenicity and better compatibility than the existing exogenous RNA vectors. Notably, the exosomes secreted by cancer cells own inherent homing ability because of their surface integrins, which can guide siRNA or miRNA through the natural membrane barrier for specific sites and effective gene delivery without any immune response. Exosomes also overcome many shortcomings related to other lipid or polymer nano-vectors, such as short cycle time, lipid toxicity, and poor stability. Therefore, exosomes secreted by cancer cells are ideal gene delivery vectors for cancer therapy Zhao et al. (2020). Identified/extracted lung-targeting exosomes from autologous triple-negative breast cancer (TNBC) cells, and developed siS100A4-loaded PMs-derived MBNPs to improve the targeted delivery to the niche before lung metastasis. The NPs perform good biocompatibility, high lung targeting ability and effectively prevent the degradation of siS100A4. Compared with conventional lipid vectors, PMs-derived MBNPs showed significant gene silencing and inhibited tumor growth significantly.

In addition, the ability of PMs-derived MBNPs can be further enhanced by adding appropriate targeting groups. Huang et al. (2022b) combined exosomes with lncRNA by engineering technology, to construct PMs-derived MBNPs (cRGD-Exo-MEG3) modified by c (RGDyK) and loaded with MEG3 for targeted therapy of osteosarcoma. The results showed that cRGD-Exo-MEG3 could deliver MEG3 to osteosarcoma cells more effectively than unmodified exosomes and equip significant anti-osteosarcoma effect. This study confirmed that the functional modification of exosomes could promote the delivery efficiency of nucleic acid molecules.

#### 4.3.6 Bacteria membranes

BM is also one of the most popular biomaterials for constructing biomimetic NPs because numerous related components on the surface that can stimulate or promote tumor-specific enrichment. Among them, outer membrane vesicles (OMVs) are the common biomimetic vectors, with diameters ranging from 10–300 nm, which are usually released by living bacteria and are the medium that enables bacteria to interact with the environment. Currently, OMVs have been proved to be related to many biological functions of bacteria, including intercellular DNA transfer, iron absorption and drug resistance (Huang et al., 2020). The surface of OMVs is rich in lipopolysaccharide and outer membrane protein, which can be recognized and absorbed by antigen-presenting cells. OMVs, as a natural adjuvant, can be sensed by cytoplasmic caspase-11 and finally secretes IL-1β to mediate immune response by binding to toll-like receptor 4 on the cell surface. Therefore, OMVs possess the ability to induce long-term anti-tumor immune responses. As the gene delivery vectors for tumor therapy, OMVs can combine immunotherapy with gene silencing to establish a strategy to eradicate malignant tumors completely. For example, Li et al. (2022) constructed an mRNA delivery platform based on OMVs, which were decorated with mRNA binding protein L7Ae and lysosome escape protein listeriolysin O (OMVs-LL-mRNA). OMVs-LL-mRNA can achieve rapid adsorption of mRNA antigen and lysosome escape, and show a significant inhibitory effect on melanoma with a complete tumor elimination tare of 37.5%. In addition, the immune memory effect established by OMVs-LL-mRNA can prevent tumor recurrence 60 days after drug withdrawal.

Moreover, OMVs can inhibit tumor growth or activate tumor immunity by regulating the metabolism of tumor cells in TME. Guo et al. (2021) prepared a pH-sensitive BMs-derived MBNPs (siRNA/PTX@OMVs) via OMVs co-loaded paclitaxel and DNA damage response 1 (Redd1)-siRNA. SiRedd1/PTX@OMVs are used to regulate development, metabolic TME and inhibit tumor growth. After releasing PTX triggered by tumor pH (pH 6.8), siRedd1/PTX@OMVs are then absorbed by M2 macrophages to increase glycolysis levels, which equip great potential in tumor-associated macrophage repolarization, tumor inhibition, tumor immune activation and TME remodeling of TNBC model. Applying BMs-derived MBNPs provides an idea for establishing co-drug delivery platforms for chemical and genetic drugs.

Notably, based on the interaction of outer membrane protein A (OmpA) with gp96 (also known as GRP94) on blood-brain barrier (BBB) endothelial cells, Gram-negative *Escherichia coli* K1 (EC-K1) can bind to BBB endothelial cells, and subsequently invade and cross BBB, colonizing the brain and ultimately inducing bacterial meningitis inflammation. In addition, other outer membrane proteins (e.g., NlpI and IbeA) can also promote EC-K1 invasion of BBB endothelial cells. Therefore, the EC-K1 outer membrane possesses the potential to construct a brain-targeted system for drug delivery and release. Inspired by this, Chen et al. (2022a) established BMs-derivedMBNPs (dOMV@NPs), which can cross the BBB into the brain via transcellular vesicle transport pathways, relying on the interaction of OmpA and gp96 between BBB endothelial cells and OmpA-mediated endosomal escape. Experimental results show that dOMV@NPs can achieve a long cycle and improve the distribution of intracranial space without significant concomitant toxicity.

#### 4.3.7 Hybrid membranes

It is found that the HMs obtained by the fusion of different biofilms can integrate the advantages of the parent membrane. For example, the HMs of erythrocytes and platelets are characterized by prolonging the blood circulation time and binding to the pathogen (Huang et al., 2023a); the HMs that fuse erythrocytes with cancer cells are endowed with the ability to prolong the half-life and homologous targeting (Chen et al., 2021a). In view of the advantages, HMs-derived MBNPs have been widely explored in tumor gene therapy. Evers et al. (2022) prepared HMs-derived MBNPs based on extracellular vesicles (EVs) and liposomes, to compensate for the defect of loading exogenous drugs or unsatisfactory gene delivery efficiency when using EVs or lipid NPs alone. The results show that HMs-derived MBNPs not only retain the original functional properties for retaining the surface markers of EVs, but can also efficiently transfer siRNA to different tumor cells. Moreover, compared with single membrane NPs, HMs-derived MBNPs significantly improved the efficiency of cell targeted uptake and gene silencing.

**FIGURE 7:**
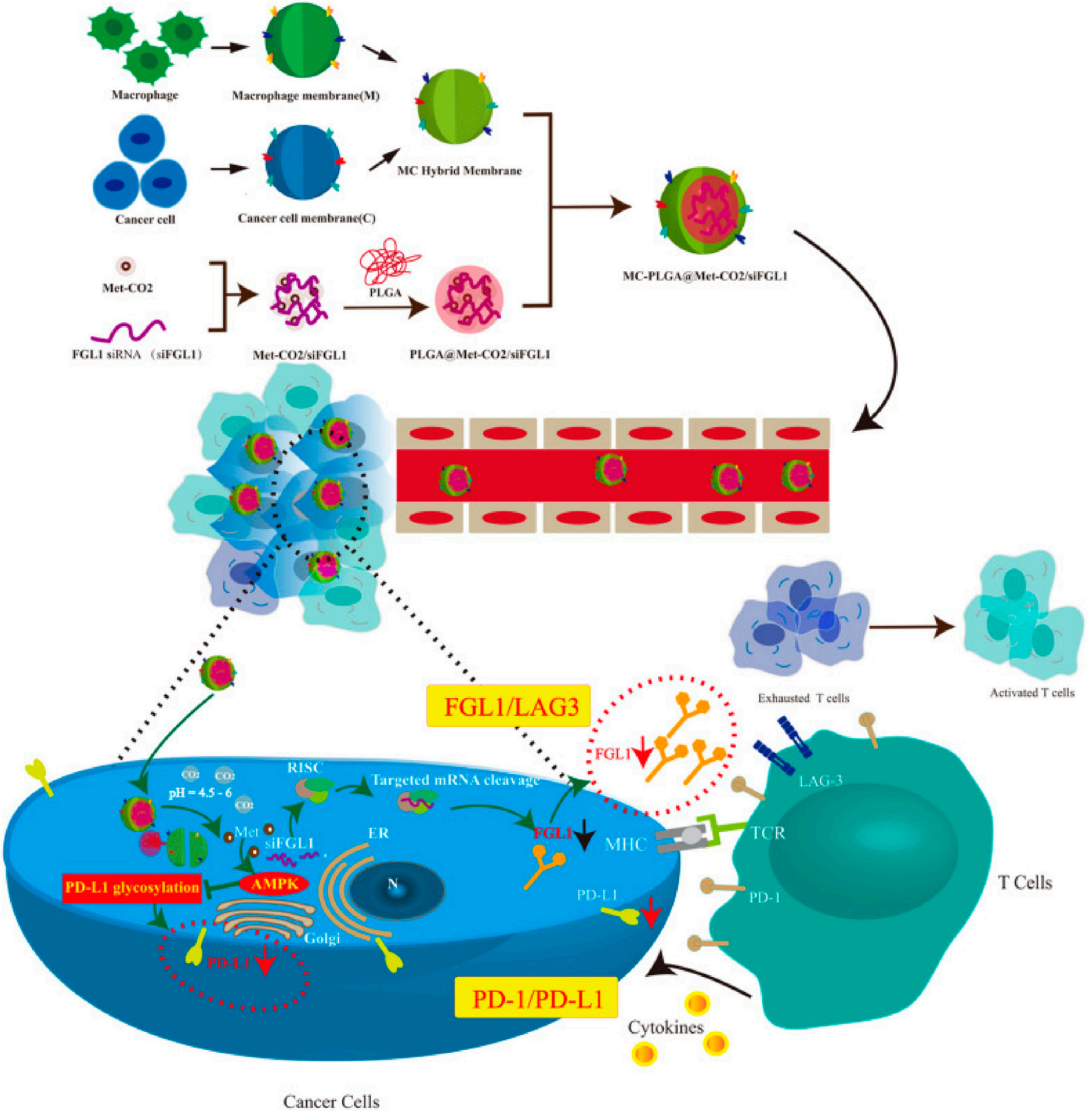
Synthesis and intracellular uptake of metformin and FGL1 siRNA-loaded pH-responsive HMs-coated NPs (Gong et al., 2021a; Huang et al., 2023c).

Moreover, overexpression of sterol regulatory element-binding protein1 (SREBP1) can promote prostate cancer’s growth and bone metastasis. SREBP silencing can inhibit PI3K/AKT signal, thus increasing the sensitivity of tumor cells to drugs. Accordingly, Chen et al. (2020b) prepared HMs-derived MBNPs (BP@siR) for siSREBP delivery by utilizing the fusion membrane of bone marrow MSCs and prostate cancer cells. In BP@siR, prostate CCMs can increase the targeting of NPs to homologous tumors, while bone marrow MSCs membrane can promote the return of NPs to bone marrow for treating tumor bone metastasis. The results showed that BP@siR could significantly downregulate the mRNA and protein levels of SREBP1 and SCD1, specifically inhibit the growth of tumors and protect the bone of the tumor site satisfactorily. In summary, HMs-derived MBNPs combine plentiful advantages of diverse biofilms and may become the future therapeutic vectors of siRNA. In addition, there are reports about other kinds of cell membranes used to construct HMs-derived MBNPs, including but not limited to EVs/liposome membranes (Evers et al., 2022; Zhou et al., 2022), CCMs/macrophage membranes (Gong et al., 2021a; Huang et al., 2023c) (Figure 7), SCMs/OMVs (Baldari et al., 2019) and CCMs/liposome membranes (Zhang et al., 2021a).

## 5 Conclusion and perspectives

In order to clearly solve the primary problems of clinical transformation and application of gene therapy, we summarized the latest progress of MBNPs to improve the efficiency of nucleic acid molecular delivery in anti-tumor therapy (Table 2). Compared with traditional vectors, MBNPs are famous for the following advantages in gene delivery: i) combine the advantages and characteristics of parent membrane and NPs, showing higher biocompatibility; ii) greatly enhance the loading capacity and stability of nucleic acid molecules *in vivo* under the action of membrane camouflage; iii) perform the extremely off-target effect for homologous targeting ability; iv) avoid the use of numerous synthetic materials and possess lower toxicity *in vivo*; v) can protect nucleic acid molecules from degradation and premature release, thus penetrating the tumor to improve the transfection efficiency; vi) the technological process is more simplified by utilizing biofilm directly, contributing to “bend overtaking” in clinical transformation application. Therefore, MBNPs exhibit significant advantages in both basic research and clinical transformation.

**TABLE 2:** Strategies for enhancing the siRNA delivery in cancer therapy of membrane-wrapped biomimetic nanoparticles.

Types	Strategies			Cancer	Cell line	Mechanisms	Ref.
	Before	NPs	Behind				
Cancer cell membranes	SiPLK1	Polymer poly (β-amino ester)	Cancer cell-like gene delivery system	Lung cancer	NCI-H1299 cell	Effective downregulation of PLK1 level; improved cellular internalization and homotypic-targeting accumulation	Zhang et al. (2020)
	siPDL	PLGA NPs	CCMs-coated nanoparticles	Breast and cervical cancers	MDA-MB-231 and HeLa cells	Facilitate the application of this strategy for effective delivery of siRNA and precise tumour therapy	Chen et al. (2019a)

	Plasmid DNA (HSVtk)	Polyethylenimine (PEI25k) NPs	PEI25k/pDNA/CM nanoparticles	Glioblastoma	C6 cells	Transfection efficiency of the nanoparticles is higher than PEI25k/pDNA complex, suggesting the homotypic targeting effect	Han et al. (2021)
	CpG-1826	PLGA NPs	Fusion cell membrane nano-vaccine	Ovarian cancer	ID8 cells	Exhibited strong immuno-activating effect both *in vitro* and *in vivo*	Zhang et al. (2022a)
	SLC7A11-targeted siRNA	None	All-in-one nanoplatform	Oral squamous cell carcinoma	CAL-27 cell	Effectively promotes the ROS accumulation and GPX4 inactivation, leading to enhanced cancer ferroptosis	Huang et al. (2022a)
	Plasmid DNA (EGFP)	Polyethylenimine NPs	CCMs-coated PEI/DNA capsules	Liver carcinoma Cervical cancer	HepG2 and HeLa cells	The internalization and transfection efficiency are 91.8% and 74.5% higher than PEI70k/DNA complexes	Liu et al. (2021)
	
Red cell membranes	miR155	Nanogel	Virus-mimicking membrane-coated miRNA nanogel	Glioblastoma	BV-2 cells	Prolongs the circulation lifetime of miR155 and endows it with active tumor-targeting capability and tumor inhibition efficacy	Gao et al. (2021)
	miR-141/200	None	RCMs-biointerface spherical nucleic acids	Lung cancer, prostatic cancer	None	Permits ultrasensitive quantification of miR-141 and show a high selectivity for discriminating miR-200 family members	Yang et al. (2019)
	SiP-gp	None	RCMs camouflaged NPs [siP-gp]	Cervical cancer	Drug-resistant HeLa cells	Enhance their physiological stability and prolong the circulation time	Xu et al. (2022)
Stem cell membranes	siPDL1	Polydopamine (PDA) NPs	SCMs camouflaged PDA NPs	Prostatic cancer bone metastases	PC-3 cells	Effectively enhance blood retention and improve accumulation, showed excellent performance in chemoimmunotherapy	Mu et al. (2021)
	SiPLK1	Fe_3_O_4_@PDA	MSCs membrane coated Fe_3_O_4_@PDA NPs	Prostatic cancer	DU145 cells	Inhibit the expression of endogenous PLK1 gene and cause obvious apoptosis in DU145 cells	Mu et al. (2018)
	Cas9/IL1RAP sgRNA	lipidoid NPs	MSCs membrane-coated nanofibril	Acute myeloid leukemia	Leukemia stem cells	Induced efficient gene editing, increased the retention time of LNP-Cas9 in the bone marrow cavity	Ho and Kim (2021)
Platelet membranes	Survivin siRNA	ZIF-8 metal-organic framework	PMs-coated MOF	Breast cancer	SK-BR-3 cells	High silencing efficiency can be achieved *in vitro* against multiple target genes	Zhuang et al. (2020)
Exosome membranes	SiS100A4	Cationic bovine serum albumin	EMs conjugated siS100A4	TNBC	4T1 cells	Protected siRNA from degradation and exhibited outstanding gene-silencing effects that significantly inhibited the growth of malignant breast cancer cells	Zhao et al. (2020)
	FOXM1 related long noncoding RNA (FRLnc1)	None	FRLnc1 in exosomes	Gastric cancer	MKN45 cells	Significantly increased the FRLnc1 expression in MKN45 cells, showed increased ability of proliferation/migration	Zhang et al. (2021b)
	lncRNA MEG3	None	c (RGDyK)-modified exosomes	Osteosarcoma	U2OS and SaOS-2 cells	Facilitate the anti-OS effects of MEG3 significantly, with the help of enhanced tumor-targeting therapy	Huang et al. (2022b)
Bacteria membranes	DNA damage response 1 (Redd1)-siRNA	None	pH-sensitive OMVs system	TNBC	4T1 cells	Provided an insight for establishing a codelivery platform for chemical drugs and genetic medicines	Guo et al. (2021)
	RNA binding protein, L7Ae	None	OMVs mRNA delivery platform	Colon cancer	MC38 colon adenocarcinoma cells	Inhibits tumor progression, elicits 37.5% complete regression, induces a long-term immune memory more than 60 days	Li et al. (2022)
Hybrid membranes	SiSREBP	Lipoic acid micelles	HMs of BMSCs and prostate cancer cells	Prostate cancer	PC-3 and C4-2B cells	Effectively suppresses tumor growth, significantly downregulated the mRNA and protein levels of SREBP1 and SCD1	Chen et al. (2020b)
	Metformin and siFGL1	PLGA NPs	Macrophages and cancer cells HMs -camouflaged PLGA NPs	Breast cancer	4T1 cells	A combination of PD-1/PD-L1 signaling blockade and FGL1 gene silencing exhibited high synergistic therapeutic efficacy against breast cancer	Gong et al. (2021a)
	Oligopeptide of eight aspartate acid (Asp8)	Bare H40-PEG nanoparticles	HMs-camouflaged NPs modified by Asp8	Oral squamous cell carcinoma	WSU-HN6 cells	Exhibited effective cancer growth inhibition properties	Chen et al. (2021a)
	SiBcl2	None	Thermosensitive liposomes with macrophage	Hepatocellular carcinoma	HepG2 cells	New system can deliver siRNA selectively and efficiently	Nai et al. (2022)
	siRNA	Lipid NPs	EVs-liposome hybrid NPs	None	Different cell types	Functionally deliver siRNA to different cell types	Evers et al. (2022)

However, there are still several obstacles that can not be ignored in the process of clinical transformation and application of the existing MBNPs for tumor gene therapy. For example, i) It is necessary to simplify the preparation method, increase the yield and reduce the cost of HMs-derived MBNPs, and maintain the original performance of the parent membrane, which developed based on a single functional membrane source. ii) The comprehensive mechanism will be revealed via in-depth basic research from individual functional proteins or receptors of biofilms, so as to make the whole process *in vivo* of MBNPs transparent, and then promote the transformation and application more widely. iii) Although MBNPs equip reassuring biosafety, the control and off-target effect of genetic molecules derived from parent membranes is still a tough challenge in clinical application. Therefore, in the production process of MBNPs, it is necessary to strictly ensure that the genetic molecules of parent cells are purified and removed, so as to avoid the introduction of additional nucleic acid molecules. A perfect MBNPs should restrain the spread/metastasis of cancer, let alone contain exogenous oncogenes/proteins. iv) In fact, researchers should pay more attention to the influence of autologous or allogeneic (immortal cell lines) biofilms on immunogenicity and manufacturability in future work. In theory, autologous cells are characterized by poor immunogenicity, but it is difficult to culture in large quantities, while allogeneic cells are the opposite. Therefore, researchers need to carefully consider the advantages and disadvantages of each membrane source and select the best parent biofilm according to the specific disease application when designing therapeutic NPs. Meanwhile, the corresponding advantages and disadvantages of various bio-membranes are also compared in Table 3. v) Finally, according to the latest report, most film coating strategies do not have a stealth effect, but at best achieve the pseudo-stealth effect (Wen et al., 2023). Therefore, the actual realization of MBNPs stealth is another major challenge faced by membrane coating technology. Instead of just adding more components, researchers should pay more attention to the overall surface structure and geometry of MBNPs, for that it is the bottleneck of NPs’ stealth performance. At present, no MBNPs officially obtained clinical research qualifications, and the reason is closely related to the above factors.

**TABLE 3:** Comparison between kinds of bio-membranes.

Types	Advantages	Disadvantages
Cancer cell membranes	Possible homologous targeting to tumor cells; tumor neo-antigens for nanovaccines	Difficult to obtain enough autologous, patient-specific cells; requires removal of all non-membrane cellular contents; possible safety concerns
Red cell membranes	Abundant in the blood and easy to separate; ease of membrane isolation; provide longer circulation; provide decoy against RBCs targeting toxins	No intrinsic targeting moieties; keep in mind the blood type used; HEME group residues potentially toxic
Stem cell membranes	Provide active targeting to solid tumors	Requires removal of all non-membrane cellular contents; complex isolation or stabilized cell lines
Platelet membranes	Relatively easy to separate; ease of membrane isolation; provide longer circulation; targeting to inflamed/damaged tissue	Less abundant in the blood
Exosome membranes	Biocompatible and biodegradable systems; low to non-immunogenic systems; protection of encapsulated nucleic acids against nucleases; decorated with tissue-specific proteins for targeted delivery; can be secreted from various cells; low liver accumulation	Not exact delivery mechanism; low yield of exosome production; low yield of loading nucleic acids into exosomes; inefficient of most purification methods; the presence of unwanted macromolecules inside the vesicles
Bacteria membranes	Allow the display of bacterial antigens without risk of active infection; antagonize bacterial adhesion to tissues	Highly antigenic material
Hybrid membranes	Simultaneously retaining the unique features of original source cells	The productive technique is not completely mature

Notably, many intracellular and extracellular physiological barriers prevent nucleic acid molecules from reaching the target of tumor cells. Therefore, on the existing basis, it is of revolutionary significance to hunt for new and more accurate biomaterials to expand the family of MBNPs, which is also regarded as the development trend of next-generation MBNPs. In particular, it is necessary to construct personalized modification and drug delivery schemes suitable for various tumors and individuals according to the complexity of the TME and the various characteristics of different tumors. For instance, the latest research shows that there is a surfactant on the surface of lung cells, which can promote the spread of drugs along the respiratory tract epithelium and enhance intracellular drug delivery. As a biomimetic material to carry nucleic acid molecules, it performs excellent advantages in local administration toward the lung (Guagliardo et al., 2021; Herman et al., 2022).

To sum up, MBNPs equip great potential as gene delivery vectors for cancer therapy. Moreover, after overcoming the challenges hindering its clinical transformation, MBNPs loaded with nucleic acid molecules will change the tumor treatment mode and harvest a broad market.
